# The Treatment of Venous Thrombosis

**Published:** 1909-11-06

**Authors:** 


### SPECIAL ARTICLE.

### THE TREATMENT OF VENOUS THROMBOSIS.

It is interesting to note the methods of treatment adopted in countries other than our own. The following is an account of the mode of procedure advocated for the treatment of ordinary phlebitis and thrombosis by Professor Albert Robin in Paris. With some of it our readers may not agree, but there are other parts of it which contain suggestions that will perhaps prove helpful.

The infective origin of all kinds of venous thrombosis is universally admitted; even phlegmasia alba dolens can be included with the rest in this respect, and although the varieties of phlebitis may differ according to the portal through which the micro-organisms gain their entrance, or according to the kind of organism at work, the degree of the mischief and its effects, these differences are more useful in making a nosological classification than in guiding therapeutic measures. Except for the general condition of the patient's organs, which may require special treatment in different cases, the medical treatment of thrombosis itself can be directed in two chief ways only—against the causal infection, and towards the repair of the lesions in the veins concerned.

### The Therapeutic Periods.

Three distinct periods can be recognised in the career of a thrombosed vein, each merging into the next without any sharp line of demarcation, and yet sufficiently distinct to merit the following names:—

I. *The acute period*, which corresponds to infection of the interior lining of the vein, the formation of clot, and the defensive reactions of the venous walls.

II. *The period of convalescence or of resolution*, during which the venous lesions undergo retrogressive changes. The blood clot becomes penetrated by budding capillaries and proliferating endothelial and connective tissue cells, which gradually absorb part of the clot, and cause the remainder to contract up against the vein-wall.

III. *The period that may be called that of residual effects*, in which the process of active retrogression has done its utmost, and yet more or less obliteration persists, with consequent venous circulatory troubles, and various deficiencies in nutrition.

In the first period, therapeutic measures must be essentially passive. They are limited to immobilising the part, and attempting to attack the offending micro-organism. In the second period, treatment is more active, since it can aid in the resolution of the blood-clot and the lesions of the vessel walls. Both physical and medicinal agencies may be employed. In the third period, therapeutic measures must be decidedly active since, without them, the residual troubles are very likely to become permanent, or at least to take a very much longer time than necessary to disappear. Here again, both physical and medicinal remedies are of use.

### 1.—Treatment during the First Period.

From the moment that phlebitis has been diagnosed, whether there is any fever or not, the affected limb must be absolutely immobilised. This may be effected conveniently by means of a splint of iron-wire netting roughly shaped like a trough or gutter to fit the thigh and leg, and then padded with wadding, the latter being kept in position by linen stitched over it. Before placing the limb in this trough splint, the leg should be covered with a thick band of wool cut in such a way that, when the limb is in position, the edges of this wool can be overlapped down the front; and the band should be long enough to extend beyond the trough-splint both above and below, and prevent the splint from pressing directly against the skin anywhere. By means of four straps and buckles applied at suitable intervals the whole may be drawn comfortably tight, immobilising the limb completely. Straps and buckles are much to be preferred to roller bandages, because the latter make it difficult to examine the part, and they cannot be removed and reapplied without a certain amount of interference with the leg.

It is well, previous to applying the splint, to sprinkle the entire limb evenly with the following powder:—

| | |
|---|---|
| Sterilised Venetian Talc (finest) ... | 80 parts |
| Boric acid ... ... ... ... | 20 parts |

Seeing that the patient cannot remain absolutely immobile for an indefinite time, and must be moved at intervals if only for the purposes of nature, it is a great convenience to have a special mechanical bed; but naturally this is out of the question in many instances in practice. A fair substitute may be improvised as follows:—Three rings can be fixed to each side of the trough-splint at suitable intervals; a double cord can then be attached to each pair of rings, the ends of the cords being adjusted and united together into a single cord at some distance from the splint; the free end of the united cords can then be passed over a pulley, either in the ceiling or in a pole fixed to the foot of the bed in much the same way as for the Hodgen's splint that is used in cases of fractured femur. The limb can be raised by means of the pulley without the least jarring, whilst one nurse, or two as the case may be, can raise the pelvis and trunk when necessary at the same time. All movements of the affected part can be avoided in this way and the chances of embolism reduced to a minimum.

### Medicinal Applications to the Part.

Is it good, at this stage, to resort to any medicinal applications for the leg? There seems little to be gained at this period from the use of mercurial ointment, iodised pomades, hot-water fomentations, compresses of ammonium chloride solution, collargol, and so forth. The putting on of any of these things necessitates some degree of movement of the part, and however gentle one may be the interference causes enough pain to counterbalance any relief that the applications themselves are likely to afford. If the pains are bearable without anything being done, put nothing on. If, on the other hand, they are unbearable, one of the most sedative of local applications seems to be:—

| | |
|---|---|
| Extract of Poppy-heads ... ... | 2 parts |
| Extract of Belladonna ... ... | 2 parts |
| Extract of Hyoscyamus ... ... | 2 parts |
| Chloroform ... ... ... ... | 10 parts |
| Simple balsamic basis ... ... | 40 parts |

It is, of course, necessary to deal with any known source of infection that may be present. If this is in the skin, antiseptic fomentations must be used, notwithstanding the above objections to local applications. If the original trouble is uterine, vaginal douches will be required; and so forth. For douches Professor Robin recommends, twice a day, three pints of sterilised water, at a temperature of 112° F., two drachms of tannic acid and 40 drops of laudanum being added to each douche.

The patient should be upon exclusively milk diet as long as there is any pyrexia; and the bowels should be carefully regulated. For the latter purpose it is much better to avoid drugs if possible and on no account should purgatives be given; at most an occasional laxative should suffice: if not, then an enema upon occasion rather than a purgative. When the temperature has returned to normal, the diet may be carefully increased by a little every day, until ordinary meals, modified according to any peculiarity presented by the patient in this respect, are reached again.

There is little or nothing to be gained by internal medication during this first stage of the phlebitis, beyond what may be required for the relief of symptoms. As long as fever is present quinine is perhaps beneficial; otherwise, medicinal treatment is purely symptomatic; malt and iron, small doses of nux vomica or hypophosphites for cachectic patients; hypnotics in the smallest doses that are found to be effective for those who do not sleep naturally; and so forth.

### The Duration of Absolute Immobilisation.

The length of time during which complete immobilisation of the part should be maintained is, upon the average, about thirty days after the last rise of temperature. In exceptional cases, such as those in which there has been no pyrexia at all, twenty-five days, or even three weeks, may be sufficient; but it should on no account be less than this under any circumstances.

There is no disadavantage in prolonging the period of immobilisation for longer than the prescribed time; the consequences of so doing are very much less troublesome than are those which follow a curtailment of the immobilisation.

It has been suggested, sometimes, that surgical treatment in phlebitis cases might have the threefold advantage of diminishing the chances of embolism, of avoiding the inconveniences of such prolonged rest in bed, and of preventing the risk of recurrence. It has sometimes been tried, and in some cases it may be good, particularly when there are large varicose veins such as should be operated upon whether they are thrombosed or not. Upon the whole, however, the evidence goes to show that surgical intervention in other cases exposes the patient to greater danger from embolism than does the above medical treatment if it is strictly carried out.

### II.—Treatment during the Second Period.

When thirty days of immobilisation have passed since the last elevation of temperature, the patient enters upon the second phase of the treatment for the condition—that of convalescence or resolution.

The first step now is to undo the pulley ropes, remove the straps and buckles, and open up the wadding which has hitherto covered the leg. The latter should remain lying in the trough splint for two days longer before any freer movement is permitted; but on the third day, if all goes well, the splint may be taken away altogether.

The limb should now be very gently and very superficially smeared with a little vaseline to remove the adherent talc; and then enclosed in a large sheet of fresh cotton wool, kept lightly in place by four straps and buckles. For the first time the leg is now lying free in the bed, and it should be left so for four days longer, unless there should be a little increase of pain in it or a slight elevation of temperature, in which case it should be left as it is until these events have entirely passed off.

The four days being gone, the cotton wool should be removed, and the limb allowed to lie in the bed with no dressing on it at all. The cleansing of the skin, begun on the day when the splint was taken away, may now be completed.

Now a big question comes up for decision, namely that of massaging and moving the limb. The researches of Cornil and Marie have shown that from the sixth day of a phlebitis onwards there is a sufficient reaction in the vein-wall to lead to the new formation of capillaries, which begin to penetrate the clot, and, along with proliferated connective tissue cells, presently convert it into a band of scar tissue.

One must not be carried away by the teaching of Marchais who declares that this process is so rapid that the phlebitis is no longer dangerous when the diagnosis has become obvious, and who attributes later embolic effects to subsequent extensions of the inflammatory processes to another segment of the vein. On the other hand, one must not fall into the opposite error, and believe with Delore and Poullet that active movements should on no account be allowed until several months of convalescence have elapsed.

The truth lies between these two extremes, and for practical purposes one may say that about six days after the splint is removed one may begin to move the leg, the patient's temperature being taken carefully and the movement being countermanded on the least rise.

The first movements should be those of flexion, extension, and circumduction of the toes, then movements of the joints of the foot and ankle, the limb not yet leaving the bed. A few days later flexion of the knee may be begun, and so on gradually until the whole leg can be lifted off the bed. If these procedures are well borne, one will begin to "educate" the veins after about ten days. The first step in this direction is to let the leg hang over the edge of the bed for a few minutes, stopping when the foot begins to be cyanosed. A little later one may begin to let the patient use his soles, by raising the head of the bed, and having a footboard for his feet to rest upon. The amount of weight bearing upon the soles can in this way be graduated to a nicety, according to the angle at which the bed is tilted up—a little more each day.

Professor Robin says of massage: " At this stage I am formally opposed to manœuvres regarded as necessary to obviate muscular atrophy, to re-awaken their contractility, to render the articulations mobile, and particularly am I opposed to procedures usually directed towards the veins themselves—that is to say, to real massage. If, however, one understands by massage those manœuvres whose particular object is to stimulate *gently* the nerve endings, the circulation and the nutrition of the limb, without any danger of disturbing the clots or of lighting up any inflammatory processes afresh, I am a firm advocate of it." That is to say, all massage at this stage should be of the gentlest. The most superficial effleurage possible should be employed, without the fingers exerting any pressure, and above all without their going over the region of the thrombus itself. This light effleurage perhaps stimulates the superficial circulation, and ultimately is of the greatest benefit to that in the deeper parts. In any case the fact to be grasped is that this manœuvre, repeated daily for from 5 to 10 minutes at a time, is of great assistance in the clearing up of the œdema and in shortening the duration of convalescence.

The duration of this period of convalescence, during which the patient should still remain in bed, varies from a fortnight to a month. The evidence that it is at an end is afforded by pressure over the affected veins being no longer painful, and by true œdema having disappeared so long as the foot is up.

### III.—The Period of Residual Effects.

Now begins the period of residual effects. The initial process in the vein is at an end, but the circulation in the limb is still more or less deficient, and the foot rapidly becomes cyanosed when it is dependent. All the muscles are below their normal size, fat has taken the place of much of their bulk, œdema comes on readily, the skin is thickened, and there are various degrees of functional inability to use the leg. The patient often experiences painful crises in the part. These are for the most part neuritic or neuralgic, and they are readily distinguished from the earlier pains along the course of the veins in that pressure over the veins themselves causes no pain. This distinction is important, for if the pains are venous, it is essential that one should go back a step and leave off both effleurage and movement; whereas if their origin is neuralgic they will disappear if effleurage and movements are continued.

The treatment now consists in a combination of bathing, wrapping up, effleurage, real massage, mobilisation, and medicaments.

### Bathing.

Every day a bath should be given, the temperature of the water being adapted to the susceptibilities of the patient. Most patients prefer it quite hot. It is good to put some bran into the water, and also a packet of mixed salts resembling in composition that of the waters of Bagnoles-de-l'Orne, made up as follows:—

| | |
|---|---|
| Sodium sulphate ... ... ... | 1 part |
| Sodium chloride ... ... ... | 150 parts |
| Calcium biphosphate ... ... | 40 parts |
| Calcium sulphate ... ... ... | 5 parts |
| Chlorides of iron and manganese ... | 1 part |
| Bicarbonate of calcium ... ... | 15 parts |
| Bicarbonate of magnesium ... ... | 26 parts |
| Silicate of lithium ... ... ... | 30 parts |
| Silicate of potassium ... ... | 30 parts |
| Silicate of aluminium ... ... | 30 parts |
| Arseniate of sodium ... ... ... | 1 part |
| Gelatine ... ... ... ... | 100 parts |

The most essential of the ingredients are the silicates and the arseniate of sodium, the quantities of which are intentionally high.

The duration of the first baths should not exceed 15 to 20 minutes; later they can be gradually lengthened out to 40 minutes. After each, the patient should lie down, and if possible sleep for an hour, and then effleurage should be performed for 10 minutes. After these 10 minutes comes a further rest for half an hour, and then the patient will be able to get up and try making a few steps, stopping as soon as the foot is cyanosed or if it becomes increasingly painful.

In this way 20 to 30 medicated baths will be taken in series; they may then be interrupted for a fortnight, and then repeated as before.

The effleurage, which at first was of the lightest possible description, will be progressively augmented, always avoiding the affected veins themselves. As time goes on it will be right to change effleurage for distinct rubbing; but it is at all times unwise to employ petrissage.

Active movements now begin to replace the former passive ones, and they are indispensable in restoring tone to the shrunken muscles, in reducing the local accumulations of fat, and in helping the thickened skin to recover its former condition. At first active movements of flexion and circumduction of the thigh will be enough; step by step the number, extent and rapidity of the movements undertaken will be gradually increased, always stopping short of actual fatigue.

Whenever the patient is upon his feet he should keep moving to avoid stagnation of the blood in the veins; when he rests, he should at once elevate the limb into the horizontal position.

### Local Applications.

At this stage, moist local applications are of great service. Compresses may be used, either of water containing the mixture of salts mentioned above or of goulard water, or of a 5 or 10-per-cent. solution of ammonium chloride. The quietest resolution is obtained with the saline mixture—a teaspoonful of the salts to a pint of water. The goulard water, and especially the ammonium chloride solution, are more energetic in their action.

When the patient begins to walk, is it wise to make him wear an elastic stocking? It is certainly not essential, provided the exigencies of business do not compel him to be up and about too soon. The best course to follow is to be very leisurely in allowing the patient to be up and about, and then to leave the muscles full liberty of action. The collateral circulation establishes itself all the faster for this. The most that should be necessary as a rule is a thin woven bandage of wool, unless the cure of the case fails to become as complete as it ought to.

Internal Medication.

Drugs given by the mouth should not be neglected, though they play a minor part in the treatment. Iodide of potassium may be given along with arseniate of sodium. A tablespoonful of the following mixture may be administered twice or thrice a day after meals:—

| | |
|---|---|
| Arseniate of soda ... ... ... | 0.05 parts |
| Iodide of potassium ... ... | 5.00 parts |
| Distilled water ... ... ... | 300.00 parts |

The mixture may well be given for ten days, then stopped for ten, and repeated.

Much can be attained by spa treatment of the residual effects of thrombosis, and Professor Robin's views upon the merits of the various baths in France are briefly as follows:—

Bagnoles-de-l'Orne has, so to speak, successfully monopolised the treatment of the after effects of phlebitis; but there are several other spas which also deserve mention. The waters of Bagnoles-de-l'Orne seem to have a special action in accelerating venous circulation, and in diminishing the stasis of the blood and consequent passive engorgement of the tissues that have been, and still are, œdematous. The external bath treatment is similar to that advocated above, in addition to which a small quantity of the water is ingested daily. It acts as a diuretic of moderate strength.

Below Bagnoles-de-l'Orne there are two other spas almost equally good. These are Bagnères-de-Bigorre and Ussat. In the first the baths are derived from the waters of the Salut. In Ussat the results obtained have been excellent in many cases, but the arrangements there are far less complete than they are at Bagnoles.

Brides-les-Bains is another place where patients of the kind are successfully treated by combined internal and external hydrotherapy; and two other spas deserve mention, namely Plombières-les-Bains for phlebitis cases with an arthritic or gouty tendency, and Bourbonne-les-Bains for those who have sustained great muscular atrophy.

When the patient is very stout, with muscular weakness, hard œdema, and thickened skin like that of elephantiasis, the mud-baths of Saint Amand and of Dax are indicated.

It may perhaps seem as if the details that have been discussed are almost too minute. Experience teaches, however, that not one of them is useless, and that it is only by attending to them strictly, patiently, and carefully that the best results can be attained in the treatment of that troublesome complaint, venous thrombosis.

